# FReDoWS: a method to automate molecular docking simulations with explicit receptor flexibility and snapshots selection

**DOI:** 10.1186/1471-2164-12-S4-S6

**Published:** 2011-12-22

**Authors:** Karina S Machado, Evelyn K Schroeder, Duncan D Ruiz, Elisângela ML Cohen, Osmar Norberto de Souza

**Affiliations:** 1 LABIO - Laboratório de Bioinformática, Modelagem e Simulação de Biossistemas. PPGCC, Faculdade de Informática, PUCRS, Av. Ipiranga, 6681 – Prédio 32, Sala 602, 90619-900, Porto Alegre, RS, Brazil; 2 GPIN - Grupo de Pesquisa em Inteligência de Negócio. PPGCC, Faculdade de Informática, PUCRS, Av. Ipiranga, 6681 – Prédio 32, Sala 628, 90619-900, Porto Alegre, RS, Brazil; 3 Programa de Pós-Graduação em Biologia Celular e Molecular, Faculdade de Biociências, PUCRS, Av. Ipiranga, 6681 – Prédio 12, Bloco A, Sala 204, 90619-900, Porto Alegre, RS, Brazil

## Abstract

**Background:**

*In silico* molecular docking is an essential step in modern drug discovery when driven by a well defined macromolecular target. Hence, the process is called structure-based or rational drug design (RDD). In the docking step of RDD the macromolecule or receptor is usually considered a rigid body. However, we know from biology that macromolecules such as enzymes and membrane receptors are inherently flexible. Accounting for this flexibility in molecular docking experiments is not trivial. One possibility, which we call a fully-flexible receptor model, is to use a molecular dynamics simulation trajectory of the receptor to simulate its explicit flexibility. To benefit from this concept, which has been known since 2000, it is essential to develop and improve new tools that enable molecular docking simulations of fully-flexible receptor models.

**Results:**

We have developed a Flexible-Receptor Docking Workflow System (FReDoWS) to automate molecular docking simulations using a fully-flexible receptor model. In addition, it includes a snapshot selection feature to facilitate acceleration the virtual screening of ligands for well defined disease targets. FReDoWS usefulness is demonstrated by investigating the docking of four different ligands to flexible models of *Mycobacterium tuberculosis’* wild type InhA enzyme and mutants I21V and I16T. We find that all four ligands bind effectively to this receptor as expected from the literature on similar, but wet experiments.

**Conclusions:**

A work that would usually need the manual execution of many computer programs, and the manipulation of thousands of files, was efficiently and automatically performed by FReDoWS. Its friendly interface allows the user to change the docking and execution parameters. Besides, the snapshot selection feature allowed the acceleration of docking simulations. We expect FReDoWS to help us explore more of the role flexibility plays in receptor-ligand interactions. FReDoWS can be made available upon request to the authors.

## Background

Today’s drug development costs have an estimated average of 800 million dollars per approved drug and the time necessary to put it into the market is between 10 to 15 years [1]. Hence, there are efforts to change these numbers, for example, by reducing the timeline and costs, and increasing the quality of the candidate drugs. An important step in the process of new drug discovery is the improvement of our understanding about target receptor-ligand interactions at the molecular level [2].

The advent of molecular biology, especially genomic sciences, and the intensive use of computer simulation tools over the past years, have had a deep impact on drug discovery [3], turning possible a more rational drug design (RDD) approach [4]. RDD basically involves a four-step cycle that combines dry and wet experiments with structure information [4]. Wet experiments are the traditional assays performed *in vitro* and *in vivo* while dry experiments are performed *in silico* or on computers.

In the RDD’s first step the structure of the target receptor (hereinafter receptor is used as synonymous of macromolecule and protein) provides a starting point for direct modeling activities. During this step, the three-dimensional (3-D) structure of the receptor obtained, for example, from the Protein Data Bank (PDB) [5], is analyzed in order to identify probable binding sites. In the second step, based on such probable binding sites, a set of possible ligands (hereinafter ligand, inhibitor and small molecules have the same meaning) is selected. It corresponds to filtering compounds from a ligand database such as ZINC [6]. Subsequently, in the third step, the receptor-ligand interactions are evaluated by computer simulations using molecular docking software, of which AutoDock [7], DOCK [8], and FlexE [9] are just a few examples. Throughout this step the ligands that had the best interaction score to the receptor are selected, bought or synthesized, and then wet-experimentally tested. Finally, in the fourth step, based on the *in vitro* results, an inhibitor candidate is detected, or the process returns to the first step.

A detailed understanding of the interaction between ligands and receptors, using molecular docking simulations, constitutes the very basis of RDD [10]. It is during molecular docking that the best ligand fit into the receptor becomes available [4]. To assess the quality of the ligand fitness, a large number of evaluations are carried out to score and rank the best ligand conformation and orientation inside the receptor binding pocket. Different approaches, including force-field methods, empirical scoring functions, and knowledge-based potentials have been developed to score receptor-ligand interactions [11]. In AutoDock3.0.5 [7], for example, the force-field based scoring function is computed in terms of the estimated free energy of binding (FEB). The more negative the estimated FEB, the more effective is the ligand-receptor association.

Most docking software is capable of simulating the different conformations that the ligands can assume inside the receptor binding pocket by considering their flexibility [7,12]. As to the receptor, the state of the art docking algorithms predict an incorrect binding pose for about 50-70 % of all ligands when only a single, rigid receptor conformation is considered [12]. However, there are limitations to the use of the explicit flexibility of the receptor [12-14] due to its large number of degrees of freedom [12-16]. Moreover, from Biology we know that receptors, such as proteins, enzymes, DNA, and RNA are inherently flexible macromolecular systems [13,17] and this flexibility is often essential for their functions [13]. Macromolecules can modify their shape upon ligand binding, molding themselves to be complementary to the ligand, increasing favorable contacts and reducing adverse interactions, thus minimizing the total FEB [13].

### Receptor flexibility in molecular docking

There are a number of alternative ways to incorporate, at least in part, the receptor flexibility in molecular docking simulations (reviewed by Teodoro and Kavraki [18], Totrov and Abagyan [12], Cozzini et al. [13], Huang and Zou [15], Wong [16], Alonso et al. [17] and Chandrika et al. [19]). Some methods consider only one receptor conformation. However, there are approaches that make use of a set of receptor conformations. Receptor conformations can be determined experimentally either by X-ray diffraction or NMR experiments, or generated by computational methods such as molecular dynamics (MD) simulations [18]. Advances in experimental techniques allowed a detailed view of biological processes by accessing details of the structural properties of biological macromolecules [20]. These properties can be further meticulously investigated by computational techniques such as MD simulations [21], which simulate the motion phenomena of biological molecules in atomic detail [22]. To consider the receptor flexibility in molecular docking experiments the multiple receptor conformations can be combined on different grid forms or can be executed as a series of molecular docking experiments, each of which with a different receptor conformation.

According to Teodoro and Kavraki [18] the first use of multiple structures derived from a MD simulation for a drug design application was by Pang and Kozikowski [23]. Carlson and co-workers [24] used the structures from MD simulation to develop a receptor-based pharmacophore where binding sites conserved during the MD were combined into a dynamic pharmacophore model. Lin et al. [25,26] presented the relaxed complex scheme (RCS) to accommodate receptor flexibility in the search for correct ligand-receptor conformations. They first performed a MD simulation on the ligand-free protein and then docked ligands to snapshots obtained from the simulation. More recently, Amaro et al. [27] showed extensions and improvements to the RCS method. They applied a more rigorous characterization of local and global binding effects and improved the computational efficiency by reducing the receptor ensemble to a representative set of conformations.

Among all the available methodologies to model the explicit flexibility of the receptor we chose to use an ensemble of different receptor conformations derived from a MD simulation trajectory. We call this a fully-flexible receptor (FFR) model. Consequently, we must execute a series of molecular docking simulations considering in each one a receptor snapshot derived from the MD trajectory. Despite that docking against several structures increases the chances of finding a receptor in the right conformational state to accommodate a particular ligand [17], this methodology can be compute-intensive [12,13,15,16]. Several computer programs are used in each receptor-ligand docking simulation and to parameterize and execute all software, manually and accurately, is not a trivial task [28].

### Workflow modeling

Nowadays, workflows are not being used just for the automation of business processes [29]: they are being used successfully to automate scientific processes as well [28,30-33] . Ludäscher et al. [30] define scientific workflows as a network of analytical steps, where the output of a step is used as input for the next step, that can involve access to and querying databases, data analysis and mining, and other steps which, normally, are computationally intensive jobs executed on high performance computing platforms. According to Ludäscher et al. [34] workflow development differs from general programming mainly in that the composition and configuration of a workflow is built from pre-existing and more general-purpose components, subflows and services; a new workflow can be created by a user by modifying an existing one, or else the user can compose a new workflow using components and subflows obtained from a repository. Moreover, scientific workflows may include support for monitoring the execution of workflows in real time, recording the processing history and allocation in distributed execution environments, and managing scientific data [34]. All these features make up for a much more complete and complex framework which is arguably not available to a simple execution of interactive scripts.

Some specific tools have already been developed to model and execute scientific workflows, e.g. Kepler [30] and Taverna [31]. Kepler is an open source tool designed to implement and execute different varieties of workflows ranging from low level workflows of interest to grid engineers to analytical knowledge discovery workflows for scientists [30]. Taverna [31] was specifically developed for the design and execution of bioinformatics workflows in a structured, repeatable and verifiable mode. Oinn et al. [31] described several other scientific workflow management systems together with a table comparing some features of these tools.

There already are a number of studies applying scientific workflows to modeling and executing bioinformatics tasks and new tools or extensions of existing ones are being developed. For instance, BioMoby-based Web Services [32] allow the construction of functional workflows, defining ontology for bioinformatics data-types and a corresponding XML representation to facilitate the flow of data from different Web-based resources. Bartocci et al. [33] presents a new WfMS called BioWMS which provides a Web-based user interface for definition, execution and results management of a scientific experiment, employing an agent-oriented technology to create a distributed, concurrent, flexible, adaptive and mobile system.

### FReDoWS

Our test simulation is 3,100 ps (3,1 ns) long, with snapshots collected at every 1,0 ps. Thus, using the FFR model, it is necessary to execute 3,100 molecular docking simulations. Executing all these simulations either manually or using shell scripts is complicated and inefficient. Their results are likely not to be reproducible when executed by different users at different times. Moreover, there is the necessity to reduce the CPU demand. To address this problem, in this article we describe FReDoWS (Flexible-Receptor Docking Workflow System), a workflow-based methodology, developed to automate molecular docking simulations that make use of a FFR model. Additionally, a snapshot selection feature was implemented to reduce the dimension of FFR models so as to accelerate its application in virtual screening of ligands [6].

## Methods

### The FReDoWs architecture

**Figure 1:**
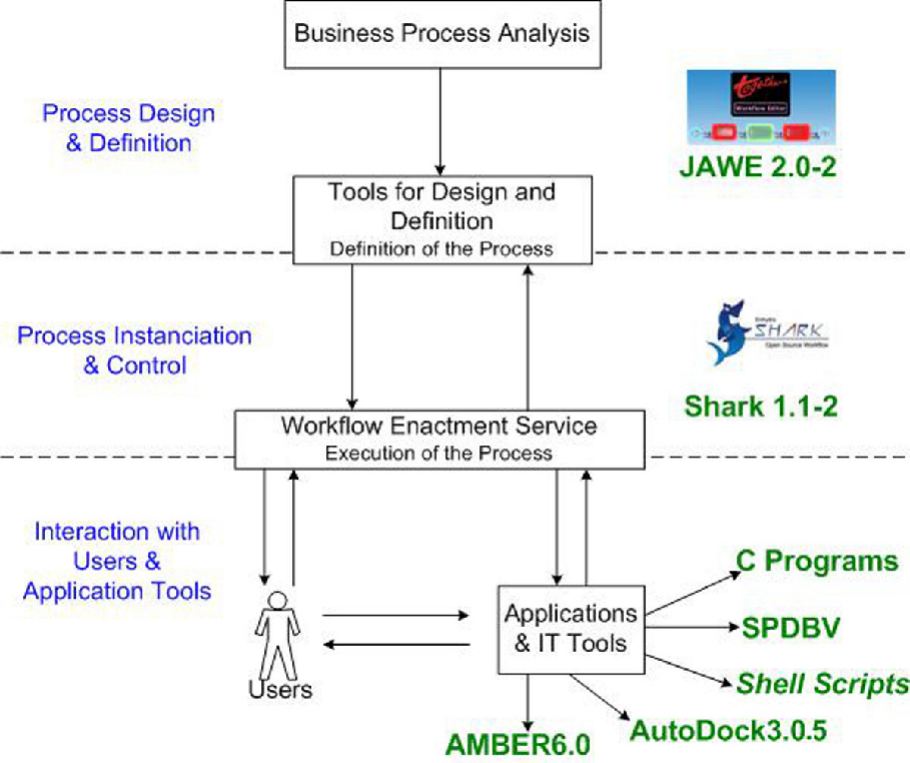
**Tools used in the development of FReDoWS.** JAWE2.0-2 is for process design and definition. Shark1.1-2 performs process instantiation and control. FReDoWS interact with several software. The Ptraj module of AMBER6.0 generates PDB files from MD simulations. This is realized with C programs and shell scripts. Swiss-PdbViewer (SPDBV) is used to visualize and manipulate receptor and ligand structures and, finally, AutoDock3.0.5 is a suite of programs to execute automated molecular docking experiments.

We adapted the WfMC [29] generic structure for a workflow to describe FReDoWS (Figure 1). Process design and definition are prepared with JAWE2.0-2 [35]. Enhydra Shark1.1-2 [36] is employed to instantiate and executes a process. During each workflow execution of docking simulation, Enhydra Shark interacts with C programs, Swiss-PdbViewer (SPDBV) [37], AutoDock3.0.5 [7] and AMBER6.0 [38] (other tools can be easily implemented through a simple modification of the workflow model).

### Process design and definition

During process design and definition we use the JAWE2.0-2 [35] (Figure 1). JAWE2.0-2 is a visual tool for creating, managing, and reviewing process definitions in a straightforward and simple manner. It allows the user to quickly create and verify workflow process definitions and store them for future use. It is also an activity-based WfMS in which processes (workflows) are comprised of activities to be completed in order to achieve a defined task [35]. JAWE has also been used in other bioinformatics workflows projects [33].

### Process instantiation and control

**Figure 2:**
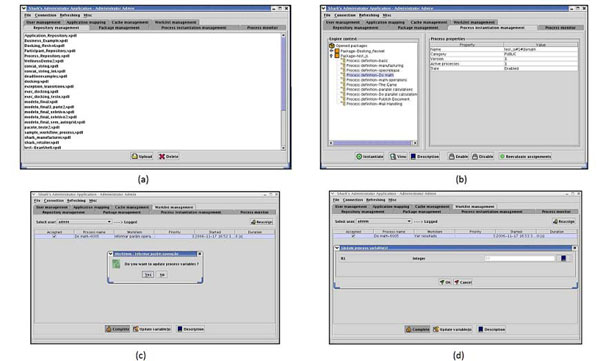
**Example of Shark’s Interface.** (a) Main Enhydra Shark interface to upload workflow models. (b) Interface in Enhydra Shark to instantiate a workflow process. (c) Example of workflow activity being executed by Shark in which it is necessary to update one variable. (d) Example of Enhydra Shark Interface to update workflow variables.

**Figure 3:**
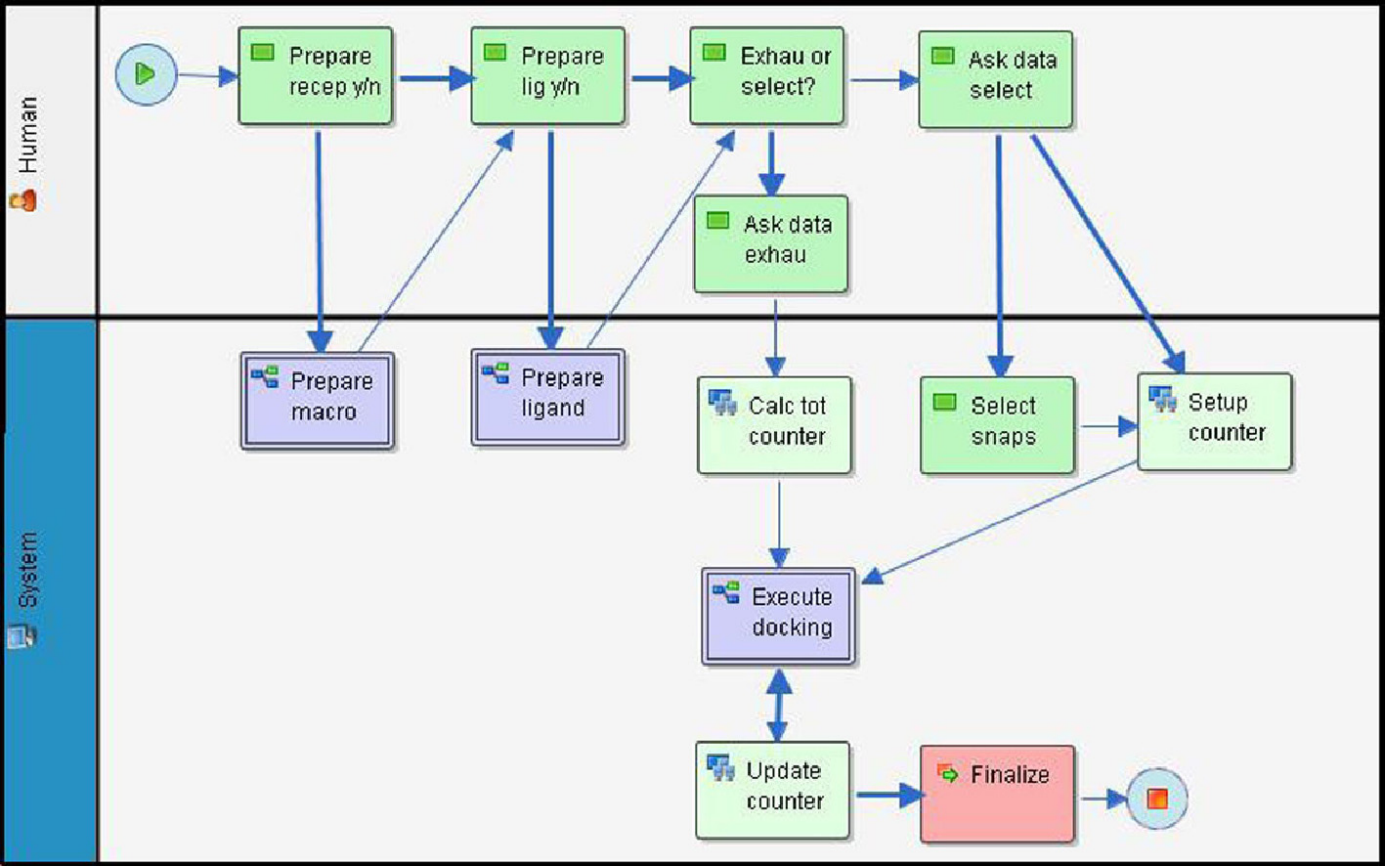
**Graphical representation of the FReDoWS model process developed in JaWE2.0-2**[35]. Each type of activity corresponds to a different color. The activities in green are executed by the user. Activities in purple indicate they are subflows, which in turn, represent a set of other activities, such as internal transitions, participants, application definitions, and other workflow relevant data. Activities in lime are executed by the system and with which the user can not interfere. This type of activity can invoke one or more external applications that are defined in JaWE and executed by Shark through a WAPI (Workflows APIs and Interchange Formats). Finally, the activity in fuchsia is used for synchronization and to formulate complex and sophisticated transitional conditions.

In the process instantiation and control, we chose to use the Enhydra Shark1.1-2 [36] as our Workflow Management System (WfMS). Enhydra Shark1.1-2 is an extendable Java Open Source workflow engine that includes a standard implementation completely based on the WfMC [29] specifications using the XPDL language. This workflow engine has implemented the WfMC "ToolAgents" API to facilitate execution of external applications as system activities. Both JAWE2.0-2 [35] and Enhydra Shark1.1-2 [36] are free software, execute in Linux and are easy to use. Figure 2a shows the main Enhydra Shark interface where the user needs to upload the FReDoWS model that was designed and defined by JAWE2.0-2. Subsequently the process is instantiated by the user as illustrated in Figure 2b. In this step the process starts to be locally executed by Enhydra Shark, with the activities released to the user according to the workflow model (Figure 3). One example is the generic activity depicted in Figure 2c. To be executed it needs to have some parameters updated by the user (Figure 2d) for the variable R1. All the FReDoWS activities are executed by C or Shell Scripts the users do not need to know or modify. They only need to set the variables for each activity, as exemplified by Figure 2d, to perform the data preparation task. The user must specify the receptor and ligand file names and their location. Afterwards, the user must set what kind of docking experiment will be executed (exhaustive or selective) and thus other variables that are needed to perform each activity or subflow.

### Application tools

During process design and definition we modeled all process activities, the majority of which needs to communicate with different application tools during each workflow execution instantiation. The tools used in FReDoWS are:

• Swiss-PdbViewer or SPDBV [37]: an application that provides a graphical user interface allowing the analysis of protein 3D structures. In our work, SPDBV is used to position the ligand in the receptor binding site during the step the ligand is prepared for the molecular docking simulation.

• AutoDock3.0.5 [7] is a suite of computer programs for molecular docking.

• AMBER6.0 [38] is a suite of programs to minimize and perform MD simulations on bio-molecules. It consists of a substructure database, a force field parameter file and also of a variety of utility programs. Ptraj, one of its modules, processes the trajectory files generated by MD simulations.

• In-house C programs and Shell scripts are used to process data during the execution of the complete workflow and to generate the results tables, for instance, by extracting information from AutoDock3.0.5 output files.

### Full receptor flexibility from molecular dynamics simulation

The receptor chosen for this study is the wild type InhA (InhA_wt) enzyme [39] from *Mycobacterium tuberculosis* and its mutants InhA_I21V and InhA_I16T, all three in a binary complex with its native ligand, the coenzyme NADH. The MD simulations of the InhA_wt-NADH, InhA_I21V-NADH and InhA_I16T-NADH complexes were performed for a time period of at least 3,100 ps [40]. The NADH coenzyme was removed from the InhA_wt and mutants’ MD trajectories for the docking experiments. Thus, three FFR models of the unliganded InhA_wt and two mutants were used in the experiments below to validate the usefulness of FReDoWS.

### Molecular docking experiments and computational infra-structure

Each FFR InhA model was submitted to a docking experiment using AutoDock3.0.5 [7]. During the executions, we used four types of computer architectures: *Cluster* is a cluster of 7 PCs Pentium III 1,0 GHz and 256 MB RAM; *PC1* is a Pentium 4 1,4 GHz and 512 MB RAM; *PC2* is a Pentium 4 2,4 GHz and 1GB RAM; *PC3* is a Core 2 Quad 2,4 GHz and 8 GB RAM.

## Results and discussion

### FReDoWS – Flexible-Receptor Docking Workflow System

FReDoWS is an acronym for Flexible-Receptor Docking Workflow System. It is a significant extension of the introductory work by Machado et al. [28], which includes a special snapshot selection feature. This feature is expected to accelerate automated docking experiments with FFR models. Figure 3 is the JaWE [35] internal representation of the FReDoWs workflow process definition. In the workflow model that we developed there are two kinds of participants: Human and System. The Human participant, represented in white (Figure 3), indicates the workflow activities that need human interference to be executed. For the System participant, an automatic agent in blue (Figure 3), activities are executed by the system without any human intervention. FReDoWS follows a task-flow model which we describe in Figure 3.

#### Setup task

Before the execution of FReDoWS there is a Setup task. This step corresponds to the generation and storing of instantaneous receptor conformations or snapshots through the execution of a MD simulation of the receptor. This task is not modeled in FReDoWS for it only needs to be executed once for each receptor.

#### Data preparation task

This task involves the preparation of receptor and ligand data. Before the execution of *Prepare macro* subflow (Figure 3), the user needs to indicate whether it is necessary to execute it in the activity *Prepare recep y/n*. This step might not be necessary if the experiment only employs a single receptor snapshot. In FReDoWS, the *Prepare macro* subflow has two activities: (1) execution of Ptraj to transform the MD trajectory files into the PDB [5] format used by almost all docking software [7-9], and (2) execution of a script to pick up snapshots separated by time intervals larger than or equal to the frequency with which snapshots are saved from the MD simulation.

The ligand data preparation is executed by the *Prepare ligand* subflow (Figure 3). As with the receptor preparation, before the execution of this subflow, the activity *Prepare lig y/n* is executed. As the *n* option shows, it may not be required to prepare the ligand if the docking experiment employs the same receptor-ligand pair (for instance when it is necessary to repeat or restart an experiment). This subflow *Prepare ligand* has two activities: (1) places the ligand in its initial position within the binding pocket of the FFR’s average structure. Receptor and ligand structures are automatically opened by Shark in Swiss-PdbViewer [37] and the ligand is manually placed by the user in the receptor active site and (2) the proper ligand file, in the Tripos Mol2 file (.mol2), is generated and transformed into the PDBQ format by the *autotors* module of AutoDock3.0.5 [7]. Since the data preparation tasks are executed by subflows, they can be easily modified to run different docking software and/or file formats through a relatively simple update of these subflows, making FReDoWS a very flexible system.

#### Execution of high-throughput molecular docking with FFR models

FReDoWS allows the user to choose the kind of docking experiments to execute. The user can run an exhaustive docking experiment [28] where the FFR model is based on the series of snapshots from a MD simulation trajectory or a selective docking experiment. In the latter, it is possible to selectively pick up particular snapshots from the MD trajectory, trimming down the dimension of the original FFR model, aiming at reducing the time necessary for each FFR-ligand docking. Therefore, when the activity *Exhau or select?* is called, the user can choose the type of workflow execution.

**Figure 4:**
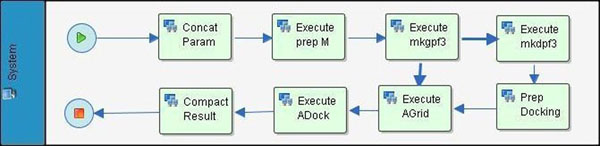
**Graphical representation of FReDoWS’ subflow *Execute docking* that executes the molecular docking simulations.** We employ the AutoDock3.0.5 [7] suite of C and AWK programs to execute docking experiments. The C programs are *addsol*, *autotors*, *autogrid* (AGrid) and *autodock* (ADock) while *mkgpf3* and *mkdpf3* are the AWK scripts. The final model of this subflow contains eight activities which execute all steps necessary for *autodock* (ADock) execution and the analyses of its output. See text for details.

In the activity *Ask data exhau* the user informs the initial and final snapshots from the MD trajectory defining the FFR model and a counter value, which designates the next snapshot to be used (the counter value, a check-pointing parameter, is a fault-tolerance feature introduced to prevent a restart from the beginning in case of workflow execution failure). Subsequently, FReDoWS executes the activity *Calc tot* counter where the total number of iterations of the subflow *Execute docking* is calculated based on the initial and final snapshots positions. Following the process definition (Figure 3), after the user indicates the type of execution, the process spawns the subflow *Execute docking* (Figure 4) where either experiment can be executed – exhaustive or selective.

The first activity, *Concat Param*, is performed to concatenate the FFR model dimension to the parameters of all following activities of this subflow. The activity *Execute prep M* prepares each individual snapshot file of the FFR model for use in AutoDock3.0.5. As a result it generates PDBQS files using the *addsol* utility. *Execute mkgpf3* and *Execute mkdpf3* generates the input.gpf and input.dpf files, respectively. These are the input files necessary for the sequential execution of *AGrid* and *ADock* . If it is the first execution, in *Prep Docking* the file input.dpf is edited within the system’s text editor and the user can effortlessly modify the docking parameters. *Execute AGrid* executes the *autogrid* module. Here grid maps are defined for each ligand atom type. *Execute ADock* runs the *autodock* module. In this step the interactions of each snapshot of FFR model and the ligand are evaluated and estimates of their affinity are calculated and expressed in terms of their free energy of binding (FEB). At the end of each docking simulation an output file is generated. It contains information about all the tested ligand positions (conformation and orientation) organized according to the best FEB and the ligand root mean square deviation (RMSD) to a reference position. *Compact Result*, the last activity executed by the subflow *Execute docking*, collects the current docking energies (FEB) and RMSDs output and stores them in a results’ list.

#### Reducing the dimension of the FFR model: the snapshot selection feature

The execution of an exhaustive experiment [28], using the FFR model of InhA enzyme [39] and the ligand molecule PIF takes about 100 hours of CPU time in the *Cluster*. Since we envisage the execution of virtual screening using FFR models of disease targets against public databases of small molecules, such as ZINC [6], containing over 13 million compounds, it is mandatory to reduce the CPU time for each docking simulation. The solution we propose is not to use the whole MD trajectory of the receptor, the FFR model, but rather a sample of snapshots from it. We call this a reduced fully-flexible receptor model (RFFR). In this work, as a preliminary approach, we use the estimated FEB and the RMSD values to reduce the dimension of the FFR model.

After an exhaustive execution of a FFR-ligand docking simulation, it is possible to execute new experiments using the same receptor model, with different ligands belonging to a single class (similar ligands), but on a selection of snapshots from the trajectory. In this way we hope to maintain the FFR model features and, at the same time, reduce CPU time. In FReDoWS, to execute a selective experiment, the user chooses the selection option in the activity *Exhau or select* (Figure 3). The following *Ask data select activity* is performed. Its input data are: *Table T0*, an output table obtained by the previously completed exhaustive experiment; *Maximum RMSD* is the value of the RMSD that the user wants to establish as one of the limiting parameters; *Total Number of Snapshots* is the number of snapshots to be selected to build the RFFR model; *Counter value* is a pointer to the start of execution. Now, the selection algorithm is ready to be executed by the activity *Select snaps*.

**Figure 5:**
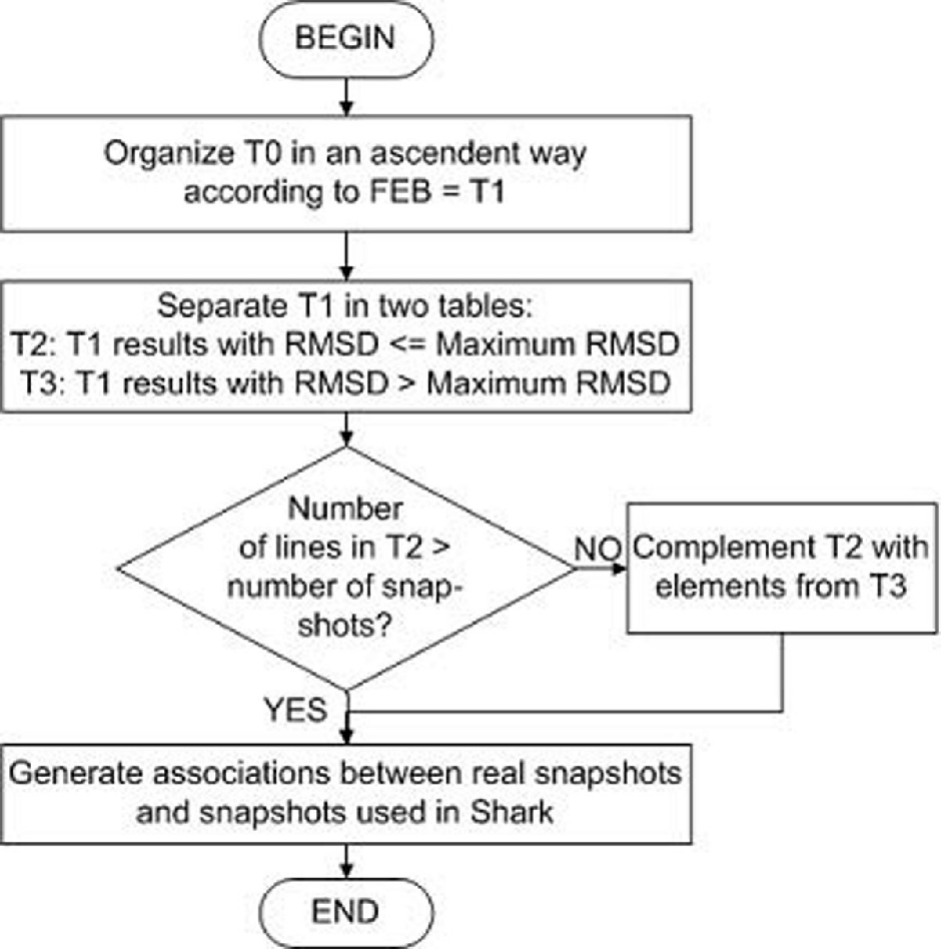
**Flowchart representation of the snapshot selection algorithm.** The algorithm is basically composed of four steps. First, the original exhaustive result table is organized in an ascendant order according to FEB. In the second step, this organized table is split in to two others tables according to RMSD values. After that, the final list of snapshots is completed and associations between real snapshots and the ones used by Shark are created.

**Figure 6:**
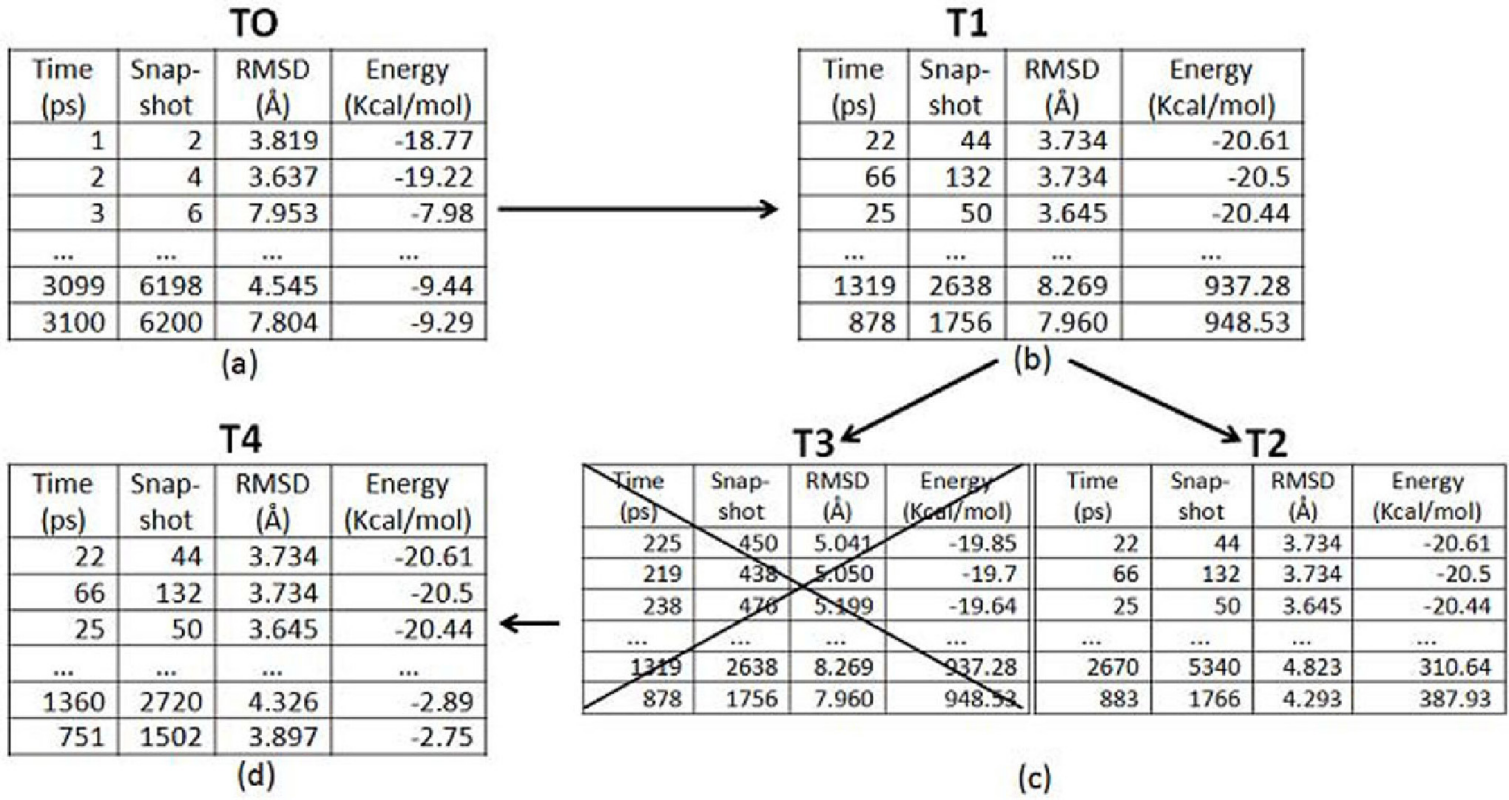
**Example of results from the snapshot selection algorithm.** (a) Table T0 of results in the order of execution from the original exhaustive docking simulations. (b) Table T1 of results assigned according to the FEB in an ascendant order. (c) T2 is the table of results in which the minimum RMSD is smaller than the Maximum RMSD specified, while T3 contains results for which the RMSD is larger than the Maximum RMSD. (d) T4 summarizes the list of receptor’s snapshots that will make up the FFR model with reduced dimension (RFFR model).

Figure 5 presents a flowchart illustrating the snapshot selection algorithm. Figure 6 shows the results for each step in the algorithm. The result of the first step in the selection algorithm is a table called *T1* (Figure 6a), that corresponds to *T0*, but organized in an ascendant order according to FEB (Figure 6b). In the second step, *T1* is separated into two tables: *T2* that corresponds to a table in which the RMSD is smaller than the *Maximum RMSD* and *T3*, which corresponds to the results with RMSD larger than the *Maximum RMSD*. In this manner we believe the selection method considers, at the same time, the best FEB values together with the results with acceptable RMSDs. In the third step, the algorithm tests if the number of rows in T2 is smaller or greater than the *Total Number of Snapshots*. If it is greater, the algorithm bypasses all steps, except the last one, because all the snapshots necessary have already been selected. But if it is smaller, it is indispensable to complete *T2* with rows from *T3*. Finally, with the snapshots selection completed (*T4 -* Figure 6d), it is now necessary to create associations between the real snapshots that are going to be used (*T4*) and the snapshots called by Enhydra Shark.

After the execution of *Select snaps* described above, the activity Setup counter is executed with the starting point of the counter assigned by the user. At last, the *Execute docking* subflow shown before is performed and all selective experiments are executed by FReDoWS.

### Test cases

The test cases are aimed at validating FReDoWS and the method for snapshots selection. We have previously executed three exhaustive docking experiments using the FFR model of InhA_wt and three ligands [28]. Therefore, in this article we describe test cases using the snapshot selection feature of FReDoWS. These tests are divided in two parts. The first part performs the selection directly from the exhaustive experiments described by Machado et al. [28]. In the second part we use only a sample of the snapshots, the RFFR model of the InhA_wt and test it against a fourth, different ligand. Finally, we describe test cases considering FFR models of InhA_wt and the mutants InhA_I21V and InhA_I16T with the ligand TCL, showing that FReDoWS can be easily applied to different FFR models and different configurations for the ligands (in these test cases we considered the TCL ligand flexible).

Before detailing these tests, we give a brief description of the enzyme receptor and its FFR model, as well as the ligands employed in the study.

#### The InhA enzyme and its FFR model

The InhA enzyme (or 2-*trans*-enoyl ACP-(CoA) Reductase, EC: 1.3.1.9) from *Mycobacterium tuberculosis* (Mtb) represents an important target for tuberculosis control [41]. Its inhibition disrupts the biosynthesis of mycolic acids, which constitute essential structures for bacterial survival [42]. Therefore, inhibitors targeting this enzyme would be promising candidates for the development of new drugs against tuberculosis [43].

InhA mutant strains have emerged almost at the same time that isoniazid (INH) was first launched in the market in the early 50's as a first line drug for the treatment of tuberculosis [44]. As an example, we have chosen to work with the mutants I21V and I16T. These mutations occur on a glycine-rich loop of InhA and were proved to be correlated to INH resistance [40].

In the search for new inhibitors, several compounds have been experimentally tested against Mtb’s InhA [45]. However, an automated method for *in silico* molecular docking and virtual screening could turn this search more extensive and effective for the identification of possible InhA ligands with improved inhibitory effects.

Schroeder et al. [40] demonstrated in a series of MD simulation studies that the InhA enzyme is a considerably flexible macromolecule and that this flexibility is reflected in its active site. Thus, we believe that for a more realistic virtual screening, this flexibility should be taken into account. For these reasons, instead of using one single, rigid, crystal structure, we use FFR models of InhA and mutants, obtained from MD simulation trajectories of the InhA-NADH complex [40].

#### The ligands NADH, PIF, TCL, and ETH

For the exhaustive test cases we used three ligands: NADH [39], PIF [46] and TCL [45]. The nicotinamide adenine dinucleotide (NADH) is the native ligand of InhA. Therefore, it was considered as a reference ligand for the docking evaluations. The NADH molecule has a total of 71 atoms. Pentacyano(isoniazid)ferrate(II) or PIF, the second ligand, contains 28 atoms. Triclosan or TCL, is a molecule with 24 atoms. Both PIF [46,47] and TCL [45] are considered good inhibitor candidates of InhA. For the selective test case we consider one ligand, Ethionamide or ETH. ETH is a small molecule composed of 21 atoms. Like isoniazid, ETH only inhibits InhA activity when covalently linked to NADH, forming the adduct ETH•NAD [44,48]. All ligands have at least one rotatable bond.

#### The exhaustive test cases

FReDoWS successfully executed three different exhaustive test cases [28] using AutoDock3.0.5 running the simulated annealing protocol with 10 runs of execution, each with 100 cycles. First the NADH ligand was docked to the FFR model of InhA_wt. The FEB and RMSD values, related to its well-known binding place [40] in InhA, were collected. The same procedure was repeated for PIF and the TCL ligands. The final results are summarized in Table 1. For simplicity, in the exhaustive test cases the ligands were considered rigid during the molecular docking simulations. However, as we show below, there is not much difference in using flexible or rigid ligands.

**Table 1:** Results of the exhaustive test cases

Ligand	Number of Atoms	Average FEB (kcal/mol)	Total Results FEB (-)	Total Results FEB (+)	Total Results Not Docked	Average RMSD (Å)	Computer Architecture	CPU Time (hs)
NADH	71	-12.9 ± 4.2	2822	278	0	5.3 ± 2.2	*Cluster*	120
PIF	28	-9.9 ± 0.6	3041	0	59	5.0 ± 1.5	*Cluster*	100
TCL	24	-8.8 ± 0.3	2836	0	264	6.8 ± 1.8	*PC1*	500

The 3rd column shows the average and standard deviation of the FEB obtained with the FFR model of InhA. The 4th column shows the total number of docking simulations that resulted in negative, favorable FEB values while the 5th column displays the unfavorable docking results with positive FEB. The 6th column presents the number of docking simulation that did not converge. The 7th column presents the average RMSD and its variation about the ligand starting position. The 8th and 9th columns show the computer architecture used and the CPU time for each FFR-ligand simulation.

For the FFR InhA-NADH complex, the docking simulation for each snapshot took about 15 minutes to complete running in the *Cluster* (see Material and Methods). Hence, it took nearly 120 hours to execute the FFR of InhA-NADH simulations. The results confirmed the affinity of the NADH ligand for the InhA enzyme, showing a good FEB average of -12.9 ± 4.2 kcal/mol. Since we are performing blind docking, i.e., we do not know a priori where the ligand should bind more efficiently in the receptor, we cannot say much about the RMSD, except that, for the FFR model, it indicates how freely the ligand moved inside its flexible binding pocket. However, as far as the FEB value is concerned, we know it is a satisfactory one, for in a previous study [40], we obtained a similar result for the average FEB using the crystallographic position of NADH as the reference ligand position to calculate the RMSD during the re-docking simulations.

The FFR InhA-PIF complex docking simulations, also executed in the *Cluster*, took around 100 hours of CPU time. The shorter CPU time is justified on the basis that PIF is significantly smaller than NADH. The FFR InhA-PIF simulations resulted in a good average FEB with low standard deviation (-9.9 ± 0.6 kcal/mol).Finally, for the exhaustive test cases, the FFR InhA-TCL docking simulations, executed in *PC1* (see Material and Methods) and took 500 hours of CPU time. The average FEB and its standard deviation (-8,8 ± 0.3 kcal/mol) indicate a favorable InhA-TCL association.

#### The selective test cases

*In silico* virtual screening for new inhibitor candidates for any target receptor molecule takes a considerable time when a single receptor structure is screened against millions or tens of millions of compounds in a database, (ZINC [6] , for instance). In a FFR model, as we [28] and others [25,26] proposed, the CPU time to span a database like ZINC should explode to an unachievable cost. Hence, the necessity to reduce the CPU time for docking simulations using a FFR model.

Given the above problem, we implemented a snapshot selection feature in FReDoWS that accelerates docking simulations which make use of FFR models derived from a MD simulation trajectory. How does the selective snapshot option of FReDoWS work? From the FFR InhA-NADH docking simulations we selected the first 1,000 best-scored and posed (lowest FEB and RMSD) docked InhA snapshots from the exhaustive FFR InhA-NADH experiment. Now, we have the RFFR model of InhA made of 1,000 snapshots only. We evaluate how good this approach is compared to the complete FFR model of InhA (composed of 3,100 snapshots). For that, we observe the difference in FEB and RMSD between the two approaches.

Three test cases were performed and divided in two parts. In the first part we simply picked up the 1,000 best-scored snapshots for the FFR InhA-PIF and FFR InhA-TCL complexes from their exhaustive docking results (Table 2) based on the selection procedure describe above. In the second part we used FReDoWS, applying the selection feature, to execute selective docking experiments with the RFFR model of InhA-ETH complex with the same docking protocol used in the exhaustive test case.

**Table 2:** Results for the selective test cases

Ligand	Number of Atoms	Average FEB (kcal/mol)	Total Results FEB (-)	Total Results FEB (+)	Total Results Not Docked	Average RMSD (Å)	Computer Architecture	CPU Time (hs)
PIF	28	-9.9 ± 0.5	1,000	0	0	4.8 ± 1.4	*-*	-
TCL	24	-8.8 ± 0.3	1,000	0	0	6.7 ± 1.7	*-*	-
ETH	21	-6.7 ± 0.3	984	0	16	5.2 ± 2.4	*PC2*	80

See Table 1 for details about the table contents.

All the selective test cases used the same 1,000 snapshots selected from the InhA-NADH results and, as the exhaustive test cases, for simplicity, considered the ligands rigid. It is worth stating, however, that the selection parameters used in this study were established for our particular experiments and that they can be modified without difficulty according to one’s requirement.

For the first part of the selective docking the FFR InhA-PIF test case had a FEB of -9.9 ± 0.6 kcal/mol and a RMSD of 5.0 ± 1.5 Å (Table 1). In the RFFR analyses we obtained a FEB of -9.9 ± 0.5 kcal/mol and a RMSD of 4.8 ± 1.4 Å (Table 2). For the FFR InhA-TCL test cases we obtained a FEB of -8.8 ± 0.3 kcal/mol and a RMSD of 6.8 ± 1.8 Å (Table 1) while for the RFFR model we obtained a FEB of -8.8 ± 0.3 kcal/mol and a RMSD of 6.7 ± 1.7 Å (Table 2). Clearly, these experiments are statistically similar; there is no significant difference between exhaustive (FFR model) and selective (RFFR model) docking test cases.

In the second part, each complete InhA-ETH docking simulation took 4 minutes of CPU time, and hence, a total execution time of 80 hours for ETH in PC2. The RFFR InhA-ETH had an average FEB of -6.7 ± 0.3 kcal/mol and a RMSD of 5.2 ± 2.4 Å. Although greater than the other FEB values, the InhA-ETH estimate of the FEB is still an acceptable one. ETH is a small ligand with only 21 atoms. Visual inspection with SPDBV [37] shows that the ETH ligand can freely move in the receptor binding pocket occupying, in most results, the proper site to form the ETH•NADH adduct that inhibits the InhA enzyme activity [44,48].

Taken together, these data suggests that our selective experiments, using the RFFR model, were successful since they were able to reproduce the FEB values for the first two ligands (PIF and TCL) using only 1,000 (RFFR model) instead of 3,100 snapshots (FFR model) of the InhA receptor and using only 1/3 of the CPU time. We also obtained accurate results for the ETH ligand, for which only the selective experiments were performed. The total CPU time for this selective experiment in a single computer was about 80 hours. If we had opted for the FFR model this time would have a three-fold increase.

Considering the analyses above, we can organize the ligands in terms of the InhA receptor affinity for them. We have found that the InhA receptor has better affinity for the NADH (its innate coenzyme ligand), followed by PIF, TCL, and ETH. From the experimental data available, we know that inhibition constant *K*_i_ for PIF is 0.086 µM [47,49] while for TCL it is 0.22 µM [45]. This means that PIF has a better inhibitory effect than TCL over the InhA receptor, corroborating our docking results. As ETH inhibits the InhA receptor as the ETH•NAD covalent adduct [50], it was expected that its FEB, as a dissociated compound, would not give a good docking result as the previous ligands.

Overall, the results obtained with the selective test cases for PIF, TCL and ETH suggests that, for these classes of ligands, the FReDoWS snapshot selection feature was effective and could be applied more extensively to other ligands to help accelerate the discovery of novel inhibitors for Mtb’s InhA.

#### The exhaustive test cases considering three FFR models of InhA

After executing the experiments with the ligands on a rigid state, a new series of experiments were performed using three FFR InhA models: the wild type (InhA_wt) and the mutants InhA_I21V and InhA_I16T aiming at comparing the binding modes in these models. MD simulation trajectories for at least 3,100 ps for each model were generated previously. These FFR models were tested against flexible TCL. The ligand flexibility was determined with the autotors module of AutoDock3.0.5 during FReDoWS execution, allowing two torsion angles.

Docking was performed using AutoDock3.0.5, genetic algorithm protocol with 25 runs, for which a maximum number of 27,000 LGA was generated on the initial population of 50 individuals, and a maximum number of 500,000 energy evaluations. The results are summarized on Table 3. Based on the standard deviations, small but relevant differences can be seen in the experiments of TCL with the different InhA receptor (wt, I21V and I16T mutants). The average FEB varied from -13.1 ± 0.5 to -11.2 ± 0.7 to -12.7 ± 0.5 kcal/mol for InhA_wt, InhA_I21V, InhA_I16T, respectively. The lowest FEB for the crystal structure was -10.8 kcal/mol.

**Table 3:** Results for the exhaustive test cases considering three FFR models of the InhA receptor

Receptor	Ligand	Average FEB (kcal/mol)	Total Results FEB (-)	Total Results FEB (+)	Total Results Not Docked	Average RMSD (Å)	Computer Architecture	CPU Time (hs)
InhA_wt	TCL	-13.1 ± 0.5	3,100	0	0	6.6 ± 2.1	*PC3*	72
InhA_I21V	TCL	-11.2 ± 0.7	3,100	0	0	5.6 ± 1.5	*PC3*	72
InhA_I16T	TCL	-12.7 ± 0.5	3,100	0	0	6.4 ± 1.3	*PC3*	72

The first column describes the receptor FFR model. The second column shows the considered ligand. See Table 1 for details about the other columns table contents.

The FEB values described in Table 3 represent a difference of about 2.5 kcal/mol when compared with the three FFR models. From this analysis we can state two basic conclusions: first, the FFR models were able to discriminate the InhA affinity for the TCL ligand. They showed that TCL binds more strongly to InhA_wt than to the mutants. Second, FFR models can accommodate a more diverse range of ligand conformations. This indicates that they are more prone to select a new ligand capable of binding to InhA than it would do if we used only a single crystal, rigid structure.

## Conclusions

The main contribution of our article is FReDoWS, a workflow-based methodology to automate the molecular docking processes using a FFR model. FReDoWS includes a snapshot selection feature to further accelerate the virtual screening of ligands for well defined disease targets. FReDoWS was developed using JAWE and Shark software tools to model and execute processes, respectively. It can be defined as a scientific workflow because it handles complex objects and files and executes complex and long-running activities. It strictly controls activities executions based on a complete workflow model, decides the next steps based on output values produced by activities and, indeed, selects different execution branches with such values.

FReDoWS usefulness was demonstrated by the investigation of the docking of four different ligands to the *Mycobacterium tuberculosis* InhA enzyme. We found that all four ligands binds effectively to the InhA FFR model as expected from the literature on similar, but wet experiments. We can also determine the extent of the receptor flexibility, and accelerate the docking screening, by performing docking simulations with selection of snapshots (the RFFR model). A work that would usually need the manual execution of many computer programs, including the docking software AutoDock3.0.5, and the manipulation of thousands of files, was efficiently and automatically performed by FReDoWS. In order to perform the same experiments without FReDoWS, one would have to be familiar with programming languages, shell scripts, command-line tools and so on. Development of these skills is time consuming and not trivial. We have demonstrated that, with a graphical user interface, the user can perform those tasks without hassling with technical details of the many tools involved. FReDoWs friendly interface allows the user to change the docking and execution parameters according to the necessity. In particular, the snapshots selection method described in this work was simple enough to illustrate the functioning of FReDoWS. As future work we will develop and analyze different snapshot selection criteria to further reduce the dimension of the FFR models, generating a much more refined RFFR while still maintaining the explicit flexibility of the receptor. We expect these advances to bring down the CPU time to a level that can allow the routine use of FFR or RFFR models in virtual screening of millions of compounds. Finally, with FReDoWS and our FFR model, defined by a collection of snapshots derived from a MD simulation trajectory, we expect to explore more of the role flexibility plays in receptor-ligand interactions.

## Competing interests

The authors declare that they have no competing interests.

## List of abbreviations

FReDoWS: Flexible-Receptor Docking Workflow System; FEB: Free Energy of Binding; FFR: Fully-Flexible Receptor; MD: Molecular Dynamics; RDD: Rational Drug Design; PDB: Protein Data Bank; RCS: Relaxed Complex Scheme; RFFR: Reduced Fully-Flexible Receptor Model; RMSD: Root Mean Square Deviation; WfMS: Workflow Management System.

## Authors' contributions

KSM implemented the scientific workflow, executed the test cases and wrote the first draft of the article. EKS and EMLC helped to conceive and execute the test cases and in the analyses of the results. DDAR helped to model the workflow from conception to execution, to conceive the test cases and to write the first draft of the article. ONS coordinates the project and wrote the final version of the article. All authors read and approved the final manuscript.
